# Policy options for tackling diet-related noncommunicable diseases

**DOI:** 10.2471/BLT.19.236356

**Published:** 2019-07-01

**Authors:** David Watkins

**Affiliations:** a Division of General Internal Medicine, University of Washington, 325 9th Ave Box 359780 Seattle, 98104 WA, United States of America.

More than four-fifths of countries (148/183 for women, 153/183 for men) are off-track to meet the health-related sustainable development goal (SDG 3) target for noncommunicable disease mortality.^1^ Trends have worsened in recent years: in more than half of countries or territories, the annual rate of decline in age-specific noncommunicable-disease mortality was slower over the period 2007–2017 than over 1990–2007.^2^ Increasing risk factor exposure, such as unhealthy diet, may be contributing to this deceleration in mortality reduction;^3^ without stronger action on major risk factors, the situation could worsen.

Against this backdrop, a new analysis in this issue of the *Bulletin* estimated the impact of voluntary targets for reducing salt, sugar and trans fatty acid intake on noncommunicable disease mortality in Portugal.^4^ While about 800 deaths could be averted annually, the probability of dying prematurely from a noncommunicable disease would only be 2–3% lower than without these voluntary targets. This percentage is far lower than the SDG 3 target of a 33% reduction in premature death from noncommunicable diseases. The study concludes that voluntary targets should be part of a broader approach that includes food labelling and excise taxes.

Implementation of the Framework Convention on Tobacco Control has demonstrated what can be achieved with a few high-value interventions.^5^ However, the success in tobacco control is probably not replicable for dietary risk factors, as the Portuguese study underscores that there are no ideal interventions for unhealthy diets. The few World Health Organization (WHO)-recommended interventions against dietary risk factors are cost–effective merely because they are cheap to implement.^6^

WHO’s list of recommended interventions against diet-related noncommunicable diseases is limited in part because the evidence generated by the scientific community has not been particularly suited for formulating and implementing nutrition policy. Researchers tend to study dietary components one at a time. However, people do not consume nutrients separately, and only in a few clinical circumstances will adjusting one or a few dietary components (for instance, iron or folate) dramatically improve health outcomes. If we continue thinking about noncommunicable disease-related dietary interventions the way we think about micronutrient deficiencies, we will continue to ask the wrong research questions.

While the recent EAT–*Lancet* Commission on healthy diets from sustainable food systems proposed a comprehensive approach to dietary risks in the context of environmental sustainability,^7^ the Commission’s recommendations have come under significant criticism on scientific grounds.^8^ Often, strong claims about the effectiveness of population-level interventions are based on weak evidence or blind spots in the science, exist. As an example of the latter, the *Lancet* Commission on Investing in Health concluded that little is known about the optimal macronutrient composition of the diet for preventing noncommunicable diseases.^9^ Improvements are needed both in the evidence base and in scientific methods for studying combinations of dietary interventions across diverse settings.

However, the results of the study by Goiana da Silva et al. in Portugal do not mean that nothing should be done about salt, sugar or trans fatty acids. The modelled reductions in intake of these dietary components were modest. Achieving larger reductions, for example through mandatory targets, could mean greater health gains.^4^ Health gains, particularly in relation to noncommunicable disease incidence and mortality, would most likely increase after 2030 as younger cohorts age.^10^ The global community can learn greatly from country experimentation with different types of policies to find out what works and what does not. Countries also create the capacity for more ambitious policies over time. For instance, a government that manages to tax sugar-sweetened beverages has built a foundation for taxing sugar in the future.^9^

Concurrently, health ministers in low-resourced countries might choose health system responses to noncommunicable diseases rather than low-impact multisectoral efforts. Packages of highly cost-effective clinical interventions against specific noncommunicable diseases like ischaemic heart disease could bring countries close to the SDG 3 target. However, the coverage of these interventions would need to be near-universal, requiring significant new investments.^9^^,^^11^ The mix of interventions that a country chooses to implement is a complex calculus influenced by many local factors; however, the Portuguese study showed that at least for now, countries should not expect much in the short-term from voluntary approaches to dietary change. A combination of taxes and strong regulations on unhealthy foods, as well as on tobacco and alcohol, and carefully selected clinical interventions will be required to achieve the SDG 3 target and sustain health gains beyond 2030.
